# Modulation of Human Motor Cortical Excitability and Plasticity by Opuntia Ficus Indica Fruit Consumption: Evidence from a Preliminary Study through Non-Invasive Brain Stimulation

**DOI:** 10.3390/nu14224915

**Published:** 2022-11-20

**Authors:** Giuditta Gambino, Filippo Brighina, Mario Allegra, Maurizio Marrale, Giorgio Collura, Cesare Gagliardo, Alessandro Attanzio, Luisa Tesoriere, Danila Di Majo, Giuseppe Ferraro, Pierangelo Sardo, Giuseppe Giglia

**Affiliations:** 1 Department of Biomedicine, Neuroscience and Advanced Diagnostics (BIND), University of Palermo, 90134 Palermo, Italy; 2 Department of Biological, Chemical and Pharmaceutical Sciences and Technologies, University of Palermo, 90134 Palermo, Italy; 3 Postgraduate School of Nutrition and Food Science, University of Palermo, 90134 Palermo, Italy; 4 Department of Physics and Chemistry “Emilio Segrè”, University of Palermo, 90134 Palermo, Italy

**Keywords:** non-invasive brain stimulation, TMS, a-tDCS, indicaxanthin, brain food, cortical excitability, homeostatic plasticity

## Abstract

Indicaxanthin (IX) from Opuntia Ficus Indica (OFI) has been shown to exert numerous biological effects both in vitro and in vivo, such as antioxidant, anti-inflammatory, neuro-modulatory activity in rodent models. Our goal was to investigate the eventual neuro-active role of orally assumed fruits containing high levels of IX at nutritionally-relevant amounts in healthy subjects, exploring cortical excitability and plasticity in the human motor cortex (M1). To this purpose, we applied paired-pulse transcranial magnetic stimulation and anodal transcranial direct current stimulation (a-tDCS) in basal conditions and followed the consumption of yellow cactus pear fruits containing IX or white cactus pear fruits devoid of IX (placebo). Furthermore, resting state-functional MRI (rs-fMRI) preliminary acquisitions were performed before and after consumption of the same number of yellow fruits. Our data revealed that the consumption of IX-containing fruits could specifically activate intracortical excitatory circuits, differently from the placebo-controlled group. Furthermore, we found that following the ingestion of IX-containing fruits, elevated network activity of glutamatergic intracortical circuits can homeostatically be restored to baseline levels following a-tDCS stimulation. No significant differences were observed through rs-fMRI acquisitions. These outcomes suggest that IX from OFI increases intracortical excitability of M1 and leads to homeostatic cortical plasticity responses.

## 1. Introduction

Non-invasive brain stimulation (NIBS) represents a gold-standard neurophysiological approach for modulating brain activity with a view to unveil potential neuronal effects of unknown substances that could implicate repercussions at synaptic, network, and cognitive levels [1,2].

Among the intriguing substances that could play a role in physiological processes, phytochemicals (PhC) have been widely investigated for their bioactive roles [3]. Nevertheless, the putative protective effects of PhC on neuronal functions represent a still debated field of research. On this point, “neuro-nutraceuticals” ascribe to a number of natural, bioactive PhC whose consumption, at nutritionally relevant amounts, is correlated with the ability to modulate neuronal processes and higher functions [4]. Only a few of them can cross the blood-brain barrier (BBB), maintain their molecular integrity, accumulate in some regions of interest, and exert a protective activity on the Central Nervous System (CNS). Polyphenols, curcumin, and berberine are just some examples of widely studied neuro-nutraceuticals able to cross the BBB [5,6,7,8]. Within this framework, specific dietary regimens comprising plant-derived compounds have been suggested to modulate neuronal excitability [9,10,11,12]. Neurophysiological measures on cortical excitability by means of TMS have been extensively collected on caffeine or caffeinated energy drinks [13,14,15,16,17], showing contrasting results. De Carvalho [17] found that a high dose of caffeine (~400 mg) caused a reduction of cortical inhibition. Moreover, research that investigated the effects of another PhC, i.e., theophylline on motor cortical excitability, found a reduction in intracortical inhibition, which is thought to depend on GABA_A_ mediated mechanisms [18]. 

*Opuntia ficus-indica* is a plant extensively cultivated worldwide for its fruits and cladodes with high content of phytochemicals and relevant anti-oxidative and anti-inflammatory properties [19,20]. In contrast to cladodes, fruits are hallmarked by a totally distinct PhC fingerprint and are enriched in the betalains pigments. Among the betalains of *Opuntia ficus-indica* fruit, indicaxanthin (IX) has been investigated over the last 20 years by the author’s research group for its chemical and nutraceutical properties [21,22]. Thanks to its reducing and amphipathic properties, the PhC modulates specific, redox-dependent signal transduction pathways, thus showing relevant redox-modulating, anti-inflammatory, anti-dysmetabolic, neuroprotective, and neuromodulatory properties [22,23,24]. In particular, the fruit supplementation was specifically found to exert anti-oxidant and anti-inflammatory effects in healthy subjects [20]. Furthermore, by demonstrating that correlations between circulating levels of inflammatory biomarkers and individual oxidative status can be uncovered in healthy individuals, these previous findings could provide an interesting topic for nutritional research on putative processes affected by these molecular properties [20]. Indeed, since inflammation and oxidative stress contribute to the alterations of physiological processes, assessing their relationships in healthy populations may help to eventually validate the effects of dietary interventions [20,23].

Potential targets underlying IX effects were identified in neuromodulation and inflammatory patterns, but also glutamatergic receptors such as AMPA and Kainate [25]. Interestingly, IX is highly bioavailable, as the ingestion of four cactus pear fruits containing 28 mg indicaxanthin generates a peak plasma concentration of 7 µM of the PhC after 3 h in humans [26]. Moreover, and in contrast with several other PhC, IX is able to cross the BBB and modulate discrete cerebral regions in the rat brain. Indeed, it has been shown that IX, orally administered to rats, modulates the in vivo bioelectric activity of hippocampal neurons, highlighting a predominantly inhibitory effect: this evidence suggested that IX could reduce neuronal excitability in this brain region [27]. 

Considering the evidence accumulated so far on the effects of IX from *Opuntia Ficus Indica* on both rodent models and healthy subjects, we aimed at investigating putative effects of IX on human cortical excitability and plasticity. To this aim, we applied paired pulse Transcranial Magnetic Stimulation (ppTMS) for assessing intracortical inhibitory and excitatory circuits of the primary motor cortex [28]. Furthermore, in order to evaluate the effect of IX on homeostatic plasticity in terms of neuronal circuit change as a consequence of given stimuli or experience, we employed anodal transcranial Direct Current Stimulation (a-tDCS), widely known to exert an excitatory effect. 

In our study, we aimed at investigating the effects induced by the ingestion of a nutritionally-relevant serving of *Opuntia Ficus Indica* yellow fruits by comparing with those obtained from the consumption of the white ones, known to be devoid of IX [29] and thus examined as placebo control. Yellow fruits were chosen according to previous data on the same cultivar, showing the highest levels of IX when compared to other cultivars [29]. To do so, we applied ppTMS and tDCS to provide insight on cortical processes that could be ascribed to underlying mechanisms of intracortical facilitation (ICF) subserved by glutamatergic circuits, short intracortical inhibition (SICI) related to GABAAergic networks [28], and neuronal excitability and plasticity, such as Long Term Potentiation (LTP) and Long Term Depression (LTD) [30]. LTP and LTD represent the paradigmatic binomial phenomena implicated in the cellular substrate of learning and memory, but also in physiopathology of different brain diseases, especially excitability-based ones [31,32].

Following in the footsteps of the human brain as a complex network, we also conducted preliminary neuroimaging evaluations to explore whether functional connectivity during the resting state (rs-fMRI) could be influenced by the consumption of the PhC present in the yellow fruits. 

All considered, our scientific investigation will attempt to add knowledge on the effects of brain stimulation on the consumption of IX-containing *Opuntia* pear fruits, which could be suggested as a source of neuro-active nutraceuticals in diet regimens in healthy subjects. 

## 2. Materials and Methods

**Figure 1:**
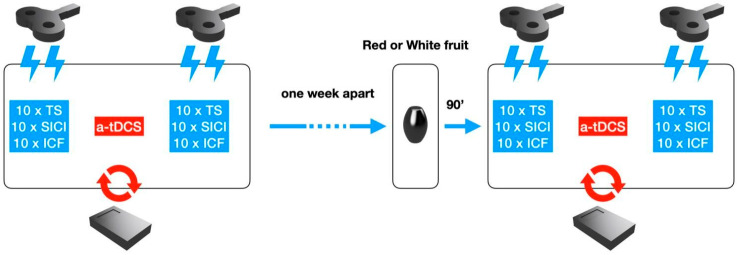
Schematic procedure of the NIBS and NIN sessions. TMS stimulation in baseline conditions was performed before and after a-tDCS to record values of Test stimulus (TS), Short Intracortical Inhibition (SICI), and Intracortical Facilitation (ICF) in random order. Next, one week apart, TMS and a-tDCS stimulations were performed 90 min after red or green fruits ingestion.

Two NIBS and non-invasive neuromodulation (NIN) sessions (baseline, post-fruit intake) per volunteer took place, separated by at least one week (as in Figure 1). Session order was randomized and balanced across subjects. 

### 2.1. Subjects

Twenty healthy male subjects, with a mean age of 20–45 years, were enrolled for the study. The volunteers were all right-handed (as measured by Edinburgh Inventory [33]: LQ > 40). They all fulfilled the requirements for NIBS and NIN, as assessed by a clinical neurologist, rigidly applying safety guidelines. According to the clinical evaluation by two experienced physicians, nobody had ongoing psychiatric disorders based on the DSM-5 criteria [34] or showed neurologic symptoms. Each volunteer underwent anamnesis questions in order to evaluate the TMS eligibility [35].

All subjects gave their written consent to the study. The experimental procedure was conducted in accordance with ethical standards of the Declaration of Helsinki (Ethical approval code: ‘Comitato Etico Palermo1_03.2022’).

### 2.2. Experimental Procedure

Our study was conceived as a single blinded and placebo-controlled design. Each subject underwent two experimental sessions. Before each session a structured interview on dietary habits was recorded and subjects were instructed to keep nutritional intake constant for all the duration of the study. In the first session (T0), 10 Test Stimulus (TS), 10 SICI, and 10 ICF were collected before and after the at-DCS session (20 min). In the second session (T1), at least one week apart, the same TMS parameters were collected 90 min after having orally assumed 400 g. of yellow cactus pear fruits (IX Group, *N* = 12) or 90 min after the consumption of white cactus pear fruits (devoid of indicaxanthin) at the same quantity (Placebo Group, *N* = 8). Four subjects had to be at last excluded from the placebo group since they did not respect the required nutritional intake during the study.

#### 2.2.1. ppTMS

Subjects were seated on a comfortable armchair, asked to be as relaxed as possible, and asked to wear a tight plastic swimmer’s cap that was later used to mark the optimal stimulation site. Electromyographic (EMG) activity was recorded by means of 0.9 cm diameter Ag-AgCl surface electrodes placed 3 cm apart with a belly and tendon montage over the right abductor pollicis brevis muscle (APB). Signals were stored with a bandpass between 10 and 1000 Hz and display gain ranging from 50 to 200 V/cm. EMG allowed a TMS simultaneous neuronal responses control, and its accurate temporary resolution was useful to verify the inhibitory or the facilitatory paired pulse provided. Nonetheless, the whole track was analyzed offline and each MEP measurement was collected per subject for subsequent statistical analyses.

The stimulating figure-of-eight coil was connected to two Magstim 200 stimulators through a Bistim module (Magstim Co., Whitland, UK). The coil was placed over the ABP- hotspot on the left primary motor cortex (Brodmann area 4) to elicit responses in the contralateral target muscle. The orientation and position of the coil were kept constant for each subject. 

The resting motor threshold (rMT) for eliciting responses in the relaxed APB muscle was defined as the intensity of stimulation needed to obtain a motor evoked potential (MEP) of at least 50 µV in at least 3 out of 6 trials [36]. The subject received audio-visual feedback of EMG activity to keep a completely relaxed state. In case of MT higher than 80% of the output, aMT (active motor threshold) was found as the minimum stimulation intensity necessary to elicit a MEP of at least 50 µV during voluntary contraction.

Once the resting MT was found, the testing stimulus and the conditioning were calculated, the former was set to 120% of the MT, the latter at an intensity of 80% of the motor threshold. ICF and SICI of primary motor cortex were assessed by means of a paired-pulse paradigm. The interstimulus interval (ISI) was 2 ms between CS and TS to record SICI, 10 ms to record ICF. The Test Stimulus (TS) was evoked alone, turning off the CS machine. The interval between each paired pulse stimulus and TS was at least 10 s. The 30 stimuli TMS set was composed by 10 SICI, 10 ICF, and 10 TS stimuli intermixed in random order [36].

#### 2.2.2. a-TDCS

In each experimental session, after the first TMS 30 stimuli set, the subject underwent anodal transcranial Direct Current Stimulation (a-tDCS) (Magstim Company Ltd., Whitland, Wales, UK). The two electrodes (35 cm^2^ (7 × 5) size), covered with saline-soaked sponges, were applied to the scalp. Anodal tDCS was delivered at 1.5 mA, with a fade-in and fade-out time of 8 s, for 20 min over the motor hotspot for the ABP muscle [37].

#### 2.2.3. Rs-fMRI Acquisition

A preliminary investigation was conducted on a dedicated sub-group of healthy subjects that also underwent evaluation of functional connectivity, following acute ingestion of yellow cactus pear fruits. Six participants were analyzed via brain MRI scan using a 1.5 T MRI unit (SignaHDxt; GE Medical System, Milwaukee, WI, USA) before and 90 min after consumption of 400 g. of yellow fruits. Acquisitions were performed through an eight-channel brain phased array coil. Structural images were obtained via a T1-weighted sagittal three-dimensional (3D) 1.2 mm thick Fast Spoiled GRadient-echo (FSPGR) prepped inversion recovery pulse sequence (acquisition matrix 256 × 256; slice thickness 1.2 mm; TR 12.4 ms; TE 5 ms; IT 450 ms; FA 20; parallel imaging method: Array coil Spatial Sensitivity Encoding, ASSET). RS-fMRI data were acquired with a two-dimensional (2D) axial T2*-weighted gradient-echo Echo-Planar (EP) pulse sequence parallel to the anterior commissure–posterior commissure (AC–PC) line over the entire brain (acquisition matrix 64 × 64; 33 slices; slice thickness 3 mm; gap 1 mm; TR 3000 ms; TE 60 ms; FA 90); the first 10 scans were discarded to allow T1 saturation to reach equilibrium; a 10-minute (200 volumes) fMRI scan was performed on each participant. The instructions provided to the subjects were the following: avoid moving during the MRI scan and quietly rest in the scanner with the open eyes and avoid thinking of anything specific. An expert neuroradiologist evaluated the images to exclude the presence of any imaging finding and artifacts that could affect the results of the rs-fMRI analyses.

Preprocessing was performed following the pipeline adopted in literature [38]. Various modules of the FMRIB’s Software Library (FSL version 5.0.9 www.fmrib.ox.ac.uk/fsl, access date 25 June 2022) were used. The analysis pipeline was composed of the following steps: motion correction, slice timing correction using Fourier-space time-series phase-shifting, spatial smoothing with a Gaussian kernel of FWHM 6.0 mm, high pass temporal filtering with sigma = 100.0 s, pre-whitening, and global spatial smoothing using a Gaussian kernel with a full width at a half maximum of 8 mm. After preprocessing, the registration of the functional images to the 2 mm isotropic MNI-152 standard space image used non-linear registration with 12 degrees of freedom. A visual quality check was guaranteed by two qualified raters to ensure correct registration.

### 2.3. Statistical Analysis

Data were analyzed by means of Repeated Measures ANOVA (rmANOVA) to compare SICI, ICF, and TEST in baseline (T0), post-yellow fruit or white fruit consumption (T1) in pre vs. post tDCS condition, by using Stat Soft Statistica (version 8.0; Dell Software, Tulsa, OK, USA). SICI and ICF were normalized and expressed as percentage of TS value. Two separate rmANOVA, with two times (T0 vs. T1) and two conditions (pre- vs. post-tDCS) as intrasubject variables in both IX and Placebo groups were performed. The post-hoc Bonferroni test was adopted to highlight potential significant differences. We considered values as statistically significant when *p* < 0.05. Data are reported as mean ± vertical bars, denoting 0.95 confidence intervals. 

As for rs-fMRI acquisition, probabilistic independent component analysis (PICA) was performed through the FSL’s MELODIC toolbox. The multi-session temporal concatenated ICA (Concat-ICA) approach was chosen. The groups of scans before and after consumption of yellow cactus pear fruits were compared to identify significantly different regions. The subjects of each couple of groups considered were concatenated for the ICA analysis. A total of 30 independent components (IC) maps were extracted. The inference on estimated maps was accomplished through a mixture model performing variance normalization, thresholding IC maps, and checking the local false-discovery rate at *p* < 0.5. The different component maps are tested voxel-wise for statistically significant differences between the groups using FSL dual regression. In particular, FSL randomized non-parametric permutation testing, with 10,000 permutations, was performed using a threshold free cluster enhanced (TFCE) technique. Correction for multiple comparisons across space was applied assuming an overall significance of *p* < 0.05 using permutation testing and TFCE.

**Figure 2:**
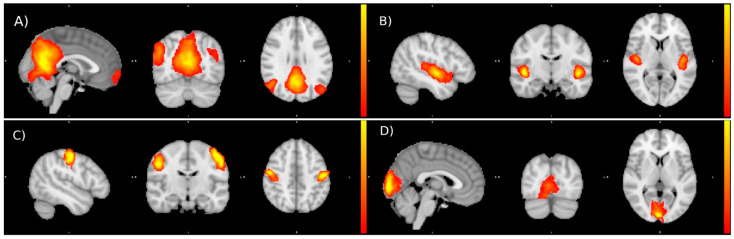
Examples of resting state networks identified by ICA: default mode network (**A**), auditory network (**B**), sensory-motor network (**C**), and visual network (**D**).

Examples of the Independent Components obtained are reported in the figure below (Figure 2):

## 3. Results

No significant harmful effects of TMS and tDCS were reported. Sixteen participants (80%) reported a slight itching sensation during the stimulation sessions, which completely subsided after a few minutes of stimulation.

### 3.1. Effects of Cactus Pear Fruit on Corticospinal Excitability (TS), Intracortical Facilitation (ICF), Intracortical Inhibition (SICI), and Cortical Plasticity (a-tDCS) in IX Group 

**Figure 3:**
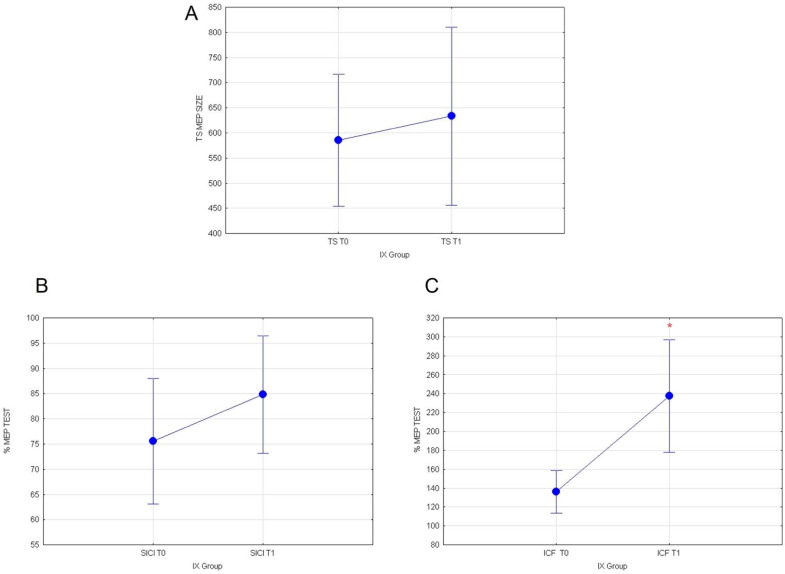
(**A**) MEP values for TEST stimulus, (**B**) normalized % MEP for SICI, and (**C**) normalized % MEP for ICF, at T0 vs. T1 for IX group are shown. Vertical bars denote 0.95 confidence intervals. (*) for *p* < 0.05.

Statistical analyses were conducted on healthy subjects following the consumption of 400 g yellow cactus pear fruits (T1) containing indicaxanthin. Our data show that the fruit ingestion is associated with the increase in intracortical excitatory circuits. Indeed, the evaluation of MEP values recorded in TEST stimulus and of normalized SICI values did not show any significant effect, respectively (F_(1117)_= 0.28; *p* = 0.59; Figure 3A) and (F_(1119)_= 1.35; *p* = 0.24; Figure 3B). Whereas, rmANOVA on normalized ICF % values recorded from subjects that assumed red fruits containing IX revealed a significant main effect of the time T0 versus T1 (F_(1119)_ = 13.75; *p* = 0.00032; Figure 3C).

**Figure 4:**
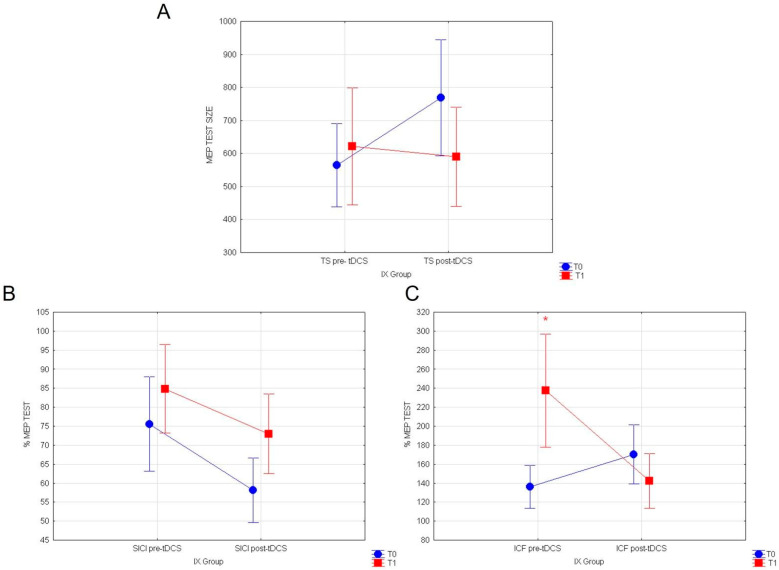
(**A**) MEP values for TEST stimulus, (**B**) normalized % MEP for SICI, and (**C**) normalized % MEP for ICF, at T0 vs. T1 for the IX group, are shown in pre vs. post-TDCS. Vertical bars denote 0.95 confidence intervals. (*) for *p* < 0.05.

Our results reveal that in the IX group, in the post-tDCS condition, the consumption of yellow fruits exerts a significant main effect on intracortical excitatory circuits, elevated by a-tDCS stimulation. In particular, a rmANOVA was conducted to compare the mean response values in T1 with two conditions (pre- vs. post-tDCS) as intra-subject variables. Neither the evaluation of TEST values outline any differences (F_(1116)_ = 2.06; *p* = 0.153; Figure 4A), nor of normalized % SICI (F_(1119)_ = 0.22; *p* = 0.63; Figure 4B) at T1 in pre- vs. post-tDCS conditions. Remarkably, statistical comparisons on normalized ICF % responses at T1 revealed a significant decreased in recorded MEP values (F_(1119)_ = 14.567; *p* = 0.00022; Figure 4C) in post-tDCS vs. pre-tDCS, following the ingestion of red fruits containing indicaxanthin. No other significant results were found.

### 3.2. Effects of Cactus Pear Fruit on Corticospinal Excitability (TS), Intracortical Facilitation (ICF), Intracortical Inhibition (SICI), and Cortical Plasticity (a-tDCS) in the Placebo Group

Statistical assessment was also carried out on healthy subjects following the consumption (T1) of 400 g white cactus pear fruits (Placebo Group) devoid of indicaxanthin. Our results show that the fruit ingestion does not modulate cortical excitability. Indeed, the rmANOVA performed on mean response values of MEP revealed no differences in T0 vs. T1, respectively, for TEST stimuli: (F_(1.79)_ = 0.85; *p* = 0.35); normalized SICI (F_(1.89)_ = 2.72; *p* = 0.10) and ICF (F_(1.89)_ = 0.76192; *p* = 0.38).

The rm ANOVA conducted on Placebo group at T1 comparing pre- vs. post-TDCS conditions outlined a significant main effect for TEST stimulus post-tDCS following white pear fruits ingestion (F_(1.79)_ = 6.17; *p* = 0.015). No significant effects were found for SICI or ICF values.

### 3.3. Effects of a-tDCS on Corticospinal Excitability (TS), Intracortical Facilitation (ICF), and Inhibition (SICI) before Assuming Cactus Pear Fruit in IX and Placebo Groups

**Figure 5:**
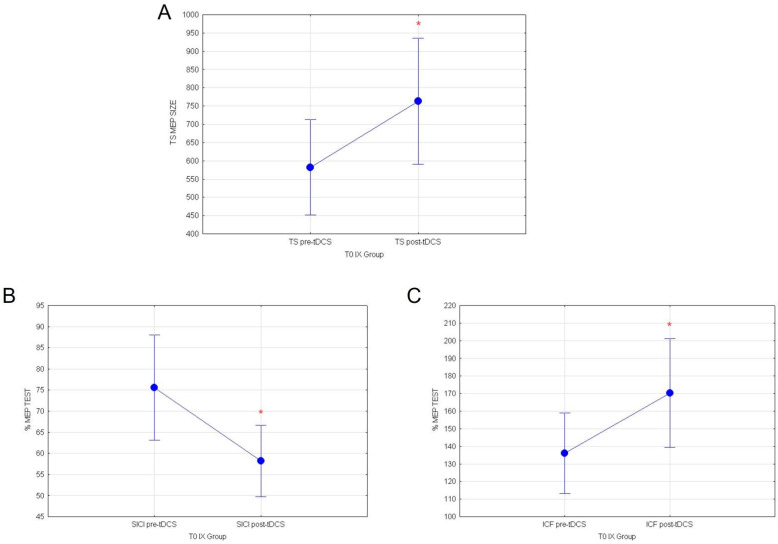
(**A**) MEP values for TEST stimulus, (**B**) normalized % MEP for SICI and (**C**) normalized % MEP for ICF, at T0 for IX group are shown in pre vs. post-TDCS. Vertical bars denote 0.95 confidence intervals. (*) for *p* < 0.05.

Statistical analyses performed on healthy subjects stimulated with a-tDCS at T0, before the ingestion of yellow cactus pear fruits (IX group), showed that a-tDCS can increase cortico-spinal excitability and intracortical GLU activity, while it inhibits intracortical GABA circuits in healthy subjects. Indeed, repeated measures ANOVA comparing the mean response values of MEP at T0 revealed a significant main effect of the condition pre vs. post-TDCS for TEST stimulus (F_(1118)_ = 3.96; *p* = 0.0487; Figure 5A). Additionally, evaluation of normalized SICI and ICF respectively showed significant reductions of intracortical inhibitory (F_(1119)_ = 5.28; *p* = 0.023; Figure 5B) and increase in excitatory circuits (F_(1119)_ = 4.00; *p* = 0.047, Figure 5C), following a-tDCS.

**Figure 6:**
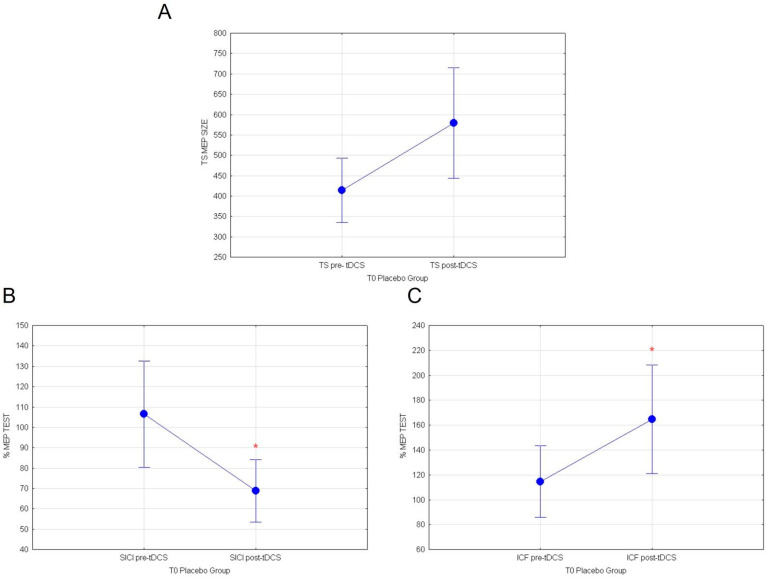
(**A**) MEP values for TEST stimulus, (**B**) normalized % MEP for SICI, and (**C**) normalized % MEP for ICF at T0 for placebo group are shown in pre vs. post-TDCS. Vertical bars denote 0.95 confidence intervals. (*) for *p* < 0.05.

Furthermore, analyses on healthy subjects stimulated with a-tDCS at T0, before the consumption of white cactus pear fruits (placebo Group), similarly showed that a-tDCS induces an increase in cortico-spinal excitability and intracortical GLU activity, concomitantly inhibiting intracortical GABA circuits. The mean response values of MEP revealed a tendency to increase in TEST stimulus following a-tDCS, which is almost significant (F_(1.79)_ = 3.85; *p* = 0.053; Figure 6A). On the other hand, rmANOVA comparing normalized SICI before white fruit ingestion pre vs. post-tDCS showed a significant inhibitory effect (F_(1.89)_ = 6.55; *p* = 0.012; Figure 6B). Finally, normalized ICF values were remarkably potentiated by a-tDCS stimulation at T0 (F_(1.89)_ = 5.13; *p* = 0.0259; Figure 6C). Significance of a-TDCS in both group are summarized in Table 1.

### 3.4. Preliminary Functional Connectivity Investigations after Cactus Pear Fruit Consumption 

Dual regression analysis on rs-fMRI datasets showed no increased resting state-functional connectivity in the six subjects that underwent MRI acquisitions before and after oral consumption of 400 g. of yellow cactus pear fruits. 

Indeed, there are no brain regions with significantly larger resting state functional connectivity after the consumption of IX-.containing fruits, considering an overall significance threshold of *p* < 0.05.

## 4. Discussion

The present work falls within the area of research on brain stimulation and neuromodulatory effects of PhC at dietary consistent amounts in healthy subjects. Here, we aim to explore the acute impact of Opuntia Ficus Indica fruit consumption on human motor cortical circuits [26]. It was indeed elucidated that IX contained in the yellow fruits is able to cross the rat BBB and exert dose-dependent effects [24,27]. In detail, brain distribution of IX was evidenced, namely in rat cortex, hippocampus, globus pallidus, thalamus and subthalamic nucleus, and neuronal modulation, which was revealed with excitatory and inhibitory effects depending on the targeted brain area. Noticeably, these findings were obtained administering an amount of the yellow betalain comparable to those measured in human plasma in volunteers that have assumed Opuntia ficus indica fruits at a dietary consumption. 

Considering the outcomes from the rodent model and from human bioavailability studies, we hypothesized that orally administered IX present in the yellow fruits could also play a role on neuronal processes in the healthy human brain. With the purpose of assessing the putative effect of IX from Opuntia Ficus Indica on basic functions of the CNS, such as cortical excitability and plasticity, we applied a neurostimulation and neuromodulation approach on the left motor cortex via TMS and a-tDCS on healthy subjects. 

We examined the difference within baseline (T0) and IX or “placebo” (T1) groups, both in pre- and in post-a-tDCS. Firstly, our results confirmed the excitatory effect induced by a-tDCS stimulation, in line with literature. Indeed, a-tDCS effects on MEP, SICI, and ICF immediately after stimulation were clearly described [39]. Additionally, evidence supports that the a-tDCS application enhances corticospinal excitability (CSE) up to 90 min, depending on the duration of a single a-tDCS session [40]. The biochemical key of a-tDCS activity could be found in the modulation of N-methyl-D-aspartate (NMDA) receptors, the main component of the glutamatergic system, and of GABA receptors, by promoting long-term potentiation LTP [41]. Symmetrically, cathodal tDCS promotes long-term depression mechanisms [42]. These LTP and LTD mechanisms can induce synaptic facilitation or depression, thus modulating synaptic efficacy. Furthermore, it was agreed that a-tDCS provides a disinhibitory after-effect corresponding to an increase in CSE, along with the reduction in SICI [43,44]. Indeed, available data on pharmacology of intracortical circuits suggest that SICI reflects the short-term postsynaptic inhibition mediated by the GABAAR, though it is still to be unveiled the exact mechanism mediated by a-tDCS. [28]. In agreement with this, we have shown specific a-tDCS effects increasing ICF and reducing SICI in basal conditions. 

Following the consumption of yellow fruits containing IX, our results revealed that IX exerted a specific role on intracortical excitability upon TMS stimulation in pre-tDCS condition. In particular, we have provided evidence that there is a major increase only in ICF values, leaving SICI unaffected. Therefore, IX contained in yellow fruits seems to achieve an excitatory drive on motor cortical excitability, corresponding to a significant raise of the intracortical facilitation, specifically subserved by glutamatergic circuits. 

Furthermore, healthy subjects were stimulated by a-tDCS after the fruit consumption and our data revealed a paradoxical effect exerted by IX-containing fruits on ICF that was reduced after anodal tDCS. This may support the idea that IX from yellow fruits induces an excitatory impact leading to homeostatic responses on motor cortical plasticity. This could be interpreted as a safety mechanism, thanks to which activity-dependent synaptic plastic changes can occur only within a physiological range. This homeostatic plasticity mechanism could play a role in neuroprotection from excitotoxicity in the context of hyperexcitability-driven diseases, known to be linked to oxidative, inflammatory, and synaptic insults [45,46].

These outcomes were further confirmed by assessing the placebo experimental group, unveiling the differences between IX-rich yellow and IX-devoid white fruits. The data obtained revealed that white fruit does not influence intracortical excitability even if it seems to increase corticospinal excitability. Although it could appear counterintuitive, a similar increase on motor cortical excitability has been reported as due to the placebo effect [47]. It seems to be specific for white pear fruit, as the yellow fruit did not affect the same parameter. A possible molecular explanation for the evidence provided could be ascribed to several receptors involved in IX-mediated effects. Its potential implication in the glutamatergic system has already been hinted at thanks to pharmacological and molecular docking approaches, showing a putative affinity with the N2A subunit of the NMDR. This kind of macromolecular complex in mammalian species is the most represented of the glutamate receptor family in hippocampus, the region where LTP was described at first [48].

Effects of other PhC on cortical excitability have been studied in the last decade. For instance, valerianic acid was found to reduce ICF, whereas no effects were reported on SICI [49]. It was also demonstrated that Hypericum perforatum modulated LTD by converting it into facilitation, via inhibition of serotonin, norepinephrine, and dopamine uptake [50,51]. Noteworthy, to the best of our knowledge, no other PhC have been proven to exert an homeostatic effect on intracortical facilitation. This could in principle play a beneficial role in disexcitability-related diseases, such migraines [31,45], or even in complex modulation of synaptic efficacy by endogenous molecular targets [52,53]. A PhC able to modulate cortical excitability could represent a safe and powerful tool to induce homeostatic responses when needed. On this point, it is worth noting that a growing body of evidence points to maladaptive or dysfunctional homeostatic plasticity as a key mechanism in many neurological and psychiatric diseases, such as Alzheimer’s Disease, Autism Spectrum Disorder [54], and depression [55]. Furthermore, diseases characterized by fluctuating cortical excitability, such as migraines [31], could take advantage of an acute treatment with IX-containing fruits. Further case-control experiments also recording behavioral data, such as motion analysis or cognitive tests, could unveil other putative effects in diseased people.

Our study also provided a preliminary evaluation of functional connectivity during resting state in healthy subjects that assumed yellow cactus pear fruits. The neuroimaging data did not identify any alterations in network connectivity. This could be due to the limited number of subjects included in the evaluation or could fit well with a specific action of IX-containing fruits that modulate intracortical circuits without modifying the physiological balance of network connectivity in acute conditions. Undoubtedly, further experiments could in future unveil eventual effects after chronic consumption, as for other PhC [56].

In the light of all this, our study paves the way to the idea that the yellow fruits containing IX could exert a neuromodulatory role on healthy subjects, and the resulting putative multi-receptorial interactions suggest conclusively an excitatory input to cortical processes. This outcome unveiling a role for IX-containing fruits in the modulation of intracortical excitation fit well with the already-evidenced neuronal effects obtained upon in vivo administration in rats. Although it should be said that IX-mediated bioelectrical effects in rats were measured in single neurons in discrete brain regions, by means of a microiontophoretic approach, here, we applied NIBS and NIN tools to investigate the overall bioelectrical activity deriving from the networks in the whole motor cortex. Notwithstanding eventual common traits, translating the preclinical study to humans allow to explore network effects due to different neuronal density, neurotransmitters, and receptorial pools circuits.

## 5. Conclusions

### Possible Limitations and Future Directions

The main limitation of our study can be found in the small sample size, even if it is to point out that the study design is based on within-subject comparisons and thus more reliable than a between-subjects design with a similar sample size. Given these pitfalls, our results should be considered as preliminary, thus further experiments with a larger series of subjects are worth being performed in order to explore alternative explanation or control for unspecific effects. Nevertheless, the lack of effect on intracortical excitability of white pear fruit seems to rule out an unspecific effect of treatment and suggest a specific modulatory role of IX present in yellow fruits that is known to exert powerful antioxidant and anti-inflammatory effects at a cellular level [20]. However, future experiments, including a different stimulation site as control condition, could strengthen conclusions on placebo effect. Indeed, also applying cathodal tDCS could add important pieces to the puzzle of red-orange cactus pear fruit effects on human motor cortical excitability, giving insight also on the putative role of Long-Term Depression (LTD) -like mechanisms.

## Figures and Tables

**Table 1:** Summary of pre-tDCS vs. post-tDCS in baseline conditions (T0) for IX and Placebo groups.

	Pre-tdcs vs. Post-tdcs
**IX Group (t0)**	
TS	*p* = 0.0487
SICI	*p* = 0.023
ICF	*p* = 0.047
**Placebo Group (t0)**	
TS	*p* = 0.053
SICI	*p* = 0.012
ICF	*p* = 0.0259

## Data Availability

Data can be requested by mail to giuditta.gambino@unipa.it.
